# Reciprocal exchange orientation to organization, challenge stressor and construal level: Three-way interaction effects on voice behavior

**DOI:** 10.3389/fpsyg.2023.1119596

**Published:** 2023-02-14

**Authors:** Long Chen, Kerrie L. Unsworth, Li Zhang

**Affiliations:** ^1^Business School, Hohai University, Nanjing, China; ^2^Leeds University Business School, Leeds, United Kingdom; ^3^School of Management, Harbin Institute of Technology, Harbin, China

**Keywords:** employees-organization reciprocal exchange orientation (EO REO), voice behavior, challenge stressor, construal level, social exchange theory

## Abstract

This study extends our understanding of voice behavior by considering a more complete set of reciprocity antecedents. We add employees-organization reciprocal exchange orientation (EO REO) into the antecedent of voice behavior and clarify the boundary condition by examining the joint moderating role of challenge stressors and construal level. The presence of challenge stressors represents a positive work environment, thus employees with a strong EO REO are likely to reciprocate with voice. However, such stressors also lead employees to focus on how to deal with the current challenges, which only aligns with employees who have a low construal level mindset and prefer to think about the details of the job at hand. Hence, we hypothesized that the positive relationship between EO REO and voice behavior in the face of challenge stressors was more likely to exist for employees whose construal level is low rather than high. We collected data from 237 employee-supervisor matched dyads in study 1 and 225 employee-supervisor matched dyads in study 2. These two studies offered support for the three-way interaction hypothesis. Our studies further voice by extending the antecedent and delineating the boundary condition of challenge stressors and construal level.

## 1. Introduction

Voice behavior is defined as a type of change-oriented extra-role behavior that facilitates organizational development (Van Dyne and LePine, 1998). Given the beneficial effect of voice behavior on employee’s creativity (Zhou and George, 2001), team performance (Sherf et al., 2018), innovation performance (Liang et al., 2019) and organization’s marketing capability (King et al., 2020), much work has been done toward identifying the antecedents that reinforce or restrict voice behavior (e.g., Detert and Burris, 2007; Ng and Feldman, 2012; Venkataramani et al., 2016; Chamberlin et al., 2017). As voice behavior is pro-social (Van Dyne and LePine, 1998), one stream of research views voice behavior as a form of reciprocation and suggests that employees who perceive their organizational environments as positive are likely to reciprocate through voice behavior (e.g., Choi, 2007; Liu et al., 2010; Zhang et al., 2014; Ng and Feldman, 2015). However, individual differences associated with the norm of reciprocity have rarely been explored in the literature of voice behavior and state-like concepts relevant to reciprocity (e.g., felt obligation) rather than trait-like concepts are the focus of these studies. Whereas state-like antecedents facilitate understanding on the proximal antecedents of the behavior (Kanfer, 1992), trait-like individual differences can offer practical implications for longer-term interventions such as training and development (Organ et al., 2010). Thus, we believe that investigating the effect of trait-like individual differences with respect to the norm of reciprocity on voice behavior will bring new knowledge to the existing literature. The present research, therefore, will examine the impact of employees-organization reciprocal exchange orientation (i.e., EO REO) which is defined as a personal belief that employees generally abide by the norm of reciprocity in exchange with their organizations (Yoshikawa et al., 2020).

In addition, empirical research observing voice behavior from a personality theory perspective has documented different findings (e.g., Crant and Wang, 2011; Nikolaou et al., 2013) and meta-analytic evidence has only reported a small relationship between personality and voice behavior (Chamberlin et al., 2017; Zare and Flinchbaugh, 2019). Moreover, previous research has reached a consensus that a more accurate prediction of behavior should consider the interaction of an individual difference with the situation (Mischel and Shoda, 1999; Barrick et al., 2013; Oreg and Berson, 2015). This means that moderators should be addressed when investigating the effect of trait-like personality on voice behavior (cf., Nikolaou et al., 2013; Li and Xu, 2020). The extant literature exploring the boundary effect of EO REO on voice behavior is relatively limited, therefore, we will explore how EO REO and situation interact in predicting voice behavior.

The situation that our present research focuses on is the presence of challenge stressors, which refers to “work-related demands or circumstances that, although potentially stressful, may have possible gains for individuals” (Cavanaugh et al., 2000, p. 68). On the one hand, challenge stressors, which include workload and time pressure (Cavanaugh et al., 2000), have become a feature of employee working that is now ubiquitous (Lu, 1997; Casey, 2012; American Psychological Association, 2018). Therefore, we believe that investigating the relationship between EO REO and voice behavior in the situation of challenge stressor has important practical implications. On the other hand, the literature of voice behavior often highlights the role of challenge stressors (e.g., Zhang et al., 2014; Chen et al., 2015; Zhou et al., 2019; Xia et al., 2020; Zhu and Wu, 2020). As employees with high EO REO strongly follow the rule of reciprocity (Yoshikawa et al., 2020) and challenge stressors are portrayed as a eustress which can bring potential benefits for employees (Cavanaugh et al., 2000), it is straightforward to assume that these employees are most likely to reciprocate *via* voice behavior when faced with such stressors.

In our study, we deepen this basic view and analyze the conditions under which challenge stressors may or may not facilitate the predictive role of EO REO on voice behavior. Accordingly, we argue that the construal level of the employee may determine whether challenge stressors moderate the relationship between EO REO and voice behavior. According to construal level theory (Vallacher and Wegner, 1989), individuals’ mental representations have different levels of construal, ranging from more concrete and detailed (lower level of construal) to more abstract and future-oriented (higher level of construal; Liberman and Trope, 1998). We argue that to acquire the potential voice-related gains associated with challenge stressors, employees need to think about how to manage the increased challenges that are occurring in the present (Cavanaugh et al., 2000; Zhu and Wu, 2020). This is more aligned with an employee who lays importance on the details of the job at hand (i.e., low construal level). Thus, we suggest that employees who have a low construal level mindset feel more comfortable in the face of challenge stressors and those who follow the norm of reciprocity will engage in positive reciprocal behavior (e.g., voice behavior). On the other hand, a high construal level mindset means that people prefer to thinking about the abstract features of the job (Liberman and Trope, 1998) and thus there is a misalignment with the challenge stressor that is causing them to focus on the details. They would therefore feel unhappy about challenge stressors and those with high EO REO may respond negatively. The above arguments make us believe that a three-way interaction will exist such that the positive relationship between EO REO and voice behavior would more likely emerge when employees with low construal level are in a situation in which challenge stressors are present.

We present two studies that examine the three-way interaction. Our studies make three contributions. First, we extend the individual difference approach in predicting voice behavior by considering the impact of EO REO. Despite personal attributes becoming the most systematic research in the literature of voice behavior (e.g., LePine and Van Dyne, 2001; Avery, 2003; Chamberlin et al., 2017; Xu et al., 2019; Li and Xu, 2020), trait-like reciprocity-related individual differences have not been explored in the voice literature. Second, it will enrich our theoretical understanding for the predictive role of reciprocity-related individual difference in the situation of challenge stressors. Most of the previous research on the personality-voice relationship have not included the situation as a moderator (e.g., Nikolaou et al., 2013; Li and Xu, 2020). This study will offer more person-in-a-situation interactionist evidence in the literature of voice behavior. Third, by introducing construal level to the moderated model, our study provides a more complete understanding of the conditional role of challenge stressors in enhancing the positive relationship between EO REO and voice behavior.

## 2. Theory and hypothesis

### 2.1. Voice behavior

Voice, as used in this study, is defined as a change-oriented communication behavior that facilitates organizational development (Van Dyne and LePine, 1998). Considerable evidence has documented the benefits of voice behavior in improving the organizational function (Guzman and Alvaro, 2019; Liang et al., 2019; King et al., 2020). As such, voice behavior has pro-social features and enables employees to make contributions to organizations. Yet voice behavior can bring potential costs for employees (Tangirala and Ramanujam, 2008). Although voice behavior can result in novel ideas that are good for the organizations it requires cognitive effort on the part of the employee, and so voice behavior potentially involves the costs of resource consumption (Liang et al., 2012). Voice behavior also incurs costs as it reveals organizational dysfunctions more directly and implies criticism of failures by stakeholders in the workplace (Liang et al., 2012). For example, Liang and Yeh’s (2019) study found that voice behavior increased the likelihood of becoming targets of workplace bullying. Therefore, voice speakers face personal risks. One way of motivating employees to take risk and speak up is to create a good working environment which contributes to cultivating employees’ willingness to repay the organization (Cropanzano and Mitchell, 2005; Liu et al., 2010; Zhang et al., 2014).

Alongside these costs, though, voice behavior can also bring some potential benefits for employees. Specifically, voice behavior is positively related to job attitudes, social status, and organizational performance (Van Dyne and LePine, 1998; Argote and Ingram, 2000; Weiss and Morrison, 2019), and therefore speaking out with constructive ideas can lead to favorable performance evaluations or even promotion opportunities (Dutton and Ashford, 1993; Thompson, 2005). It implies that employees can exchange benefits with their organizations *via* making contribution to organizational development. Accordingly, we suggest that the other way of increasing voice behavior is enhancing employees’ reciprocity belief that employees can obtain organizational recompense by participating in organizational management.

### 2.2. The contextualized relationship between EO REO and voice behavior

We believe that the two abovementioned pathways require a precondition that employees should possess the faith of reciprocity norm. Reciprocity is probably the most influential exchange norm in social exchange theory (Blau, 1964). The universal view of reciprocity norm is that one should pay back what one receives from others, and one will be punished if one does not comply (Gouldner, 1960). This underlying rule regulates REO which refers to “an employee’s belief in favor of the norm of reciprocity in exchange with other members in the workplace” (Yoshikawa et al., 2020, p. 295).

Previous research has reported that voice behavior occurs when the employee has received organizational support based on the consideration that employees should follow reciprocity norms (Van Dyne and LePine, 1998; Liu et al., 2010; Liang et al., 2012). However, the concept of EO REO tells us that not every employee believes in reciprocity (Yoshikawa et al., 2020) and only employees with high EO REO desire to repay organization when their working environment is favorable. Evidence also supports that employees’ obligation to repay organization is more sensitive to perceived organizational support when employees’ acceptance of reciprocity norm is high (Eisenberger et al., 2001). Therefore, based on this solid existing research, we assume that employees who have a strong belief in the norm of reciprocity in exchange with organizations will have a stronger tendency to speak up as a response to positive treatment by the organization than others.

Challenge stressors, which include workload, time pressure, high job responsibility and task complexity and so on, are one such form of positive treatment by the organization; they are stressful, but they also bring potential gains for the employee (Cavanaugh et al., 2000; Zhang et al., 2014). Evidence has shown that challenge stressor can lead to some beneficial outcomes such as reducing turnover intention, increasing organizational commitment and elevating work performance (Lepine et al., 2005; Podsakoff et al., 2007).

Thus, we propose that employees whose EO REO is high are willing to help organization to achieve goals when they receive organizational support or experience a positive work environment and believe they can acquire benefits from their organizations by offering help for organizations. More specifically, we argue that EO REO will be positively associated with engagement in voice behavior when they are working in a situation high in challenge stressors–the situation is one that provides an imbalance in the exchange relationship and the trait is one that urges the employee to rectify this imbalance. Therefore, we make the following hypothesis:

Hypothesis 1: The relationship between EO REO and voice behavior is positive when challenge stressors are high and non-significant when challenge stressors are low.

### 2.3. The joint moderating role of challenge stressor and construal level

Thus far our theorizing has been based on a solid platform of existing research. However, we argue that this straightforward hypothesis does not represent a true picture of employees at work because employees do not all perceive challenge stressors in the same way. According to construal level theory (Trope and Liberman, 2010), individuals’ mental representations are organized in a hierarchy, and that they vary from more concrete (lower construal) to more abstract (higher construal). Low-level construal is specific, contextualized and captures subordinate traits of targets. Conversely, high-level construal is abstract, decontextualized and related with superordinate features of targets. Construal level can be regarded as a habit with which people represent work activities (Vallacher and Wegner, 1989): people with low construal level may focus on the feasibility aspects of the job (e.g., means to an outcome; how to complete the job at hand) and those with high construal level prefer to considering its desirability aspects (e.g., the value of an action’s end state; why completing the job would be meaningful).

Using construal level theory, we can predict that construal level will moderate the role of challenge stressors in moderating the effect of EO REO on voice behavior. Lazarus and Folkman (1984) propose that the stressor which is evaluated as challenging will motivate employees to adopt an active problem-focused style of behavioral coping. When using problem-focused coping, employees would make detailed plans to deal with the problem (Lazarus and Folkman, 1984). As challenge stressors are perceived as having the potential for rewards and growth (Cavanaugh et al., 2000), it can be referred to as challenge appraisals (Lazarus and Folkman, 1984). Considerable evidence also support this premise that challenge stressor is positively related with challenge appraisal (Webster et al., 2011; Ma et al., 2021). Hence, we argue that challenge stressors require the employee to adopt the problem-focused coping style and focus on the increased challenges that are occurring. This requirement is consistent with the mindset of employees who are accustomed to thinking about the details of the job at hand (i.e., low construal level). As a result, those employees are able to cope with high challenge stressors in the way that they prefer and acquire personal growth. In this case, employees who want to follow the norm of reciprocity (i.e., high EO REO) would have a high tendency to engage in pro-organizational behavior (e.g., voice behavior) in return. Thus, when employees have low construal level, EO REO has a positive relationship with voice behavior under the condition of high challenge stressors.

In comparison, employees with high construal level prefer to consider the value of completing the job rather than think about the detailed plans of finishing the job at hand (Liberman and Trope, 1998). To cope with challenge stressors successfully, those employees must change their thinking habits to one more associated with low construal. Therefore, employees who have high construal level mindset may feel uncomfortable, because they are taken away from their preferred thinking habits under the condition of high challenge stressors. In this case, the reciprocation may be negative implying that employees with high EO REO may respond negatively and be reluctant to engage in voice behavior. Further, employees with low EO REO would not comply with the reciprocity norm in the workplace (Yoshikawa et al., 2020). Consequently, the relationship between EO REO and voice behavior may be non-significant or even negative when employees with high construal level are faced with challenge stressors. Therefore, we make the following hypothesis:

Hypothesis 2: A three-way interaction will emerge such that the positive effect of EO REO on voice behavior is stronger when challenge stressor is at high level and construal level is at a low level.

Two studies, both using employee-supervisor matched data, were used to test our hypotheses. Both studies asked employees to report EO REO, challenge stressor, and construal level while voice behavior was rated by their immediate supervisors. In total we collected data from 462 matched employee-supervisor dyads; we kept the studies separate as the two samples enabled us to test the generalizability and replicability of the findings.

## 3. Study 1

### 3.1. Participants and procedures

Participants in Study 1 were accessed with the help of Master of Business Administration (MBA) students. These MBA students were from 12 different enterprises in China involving administrative units, finance, education, manufacturing, computer and construction industry. We asked these students to produce a list of their colleagues and email addresses. We randomly selected 399 participants from this list by a random unrepeated sampling procedure, and distributed questionnaires to these participants *via* email. As participants in this study were from a wide range of sources (i.e., 12 different companies), the generalization of our findings can be improved. Each participant completed survey items measuring EO REO, challenge stressor, construal level, control variables (personal information). For each participant, his or her immediate supervisor was invited to rate the focal employee’s voice behavior. We obtained valid surveys from 237 participants who had matched immediate supervisors, and these data were included in our final analysis. No supervisors rated more than one subordinate. Among these data, over half (54.00%) of these participants were male and 38.40% were unmarried. The average age of these participants was 30.806 years (*S.E.* = *5.569 years*). In terms of education, 15.60% of the participants had master’s degrees and above, 75.90% of them had bachelor’s degree, and the others had lower levels of schooling. The average organizational tenure of the participants was 5.163 years (*S.E.* = *4.696 years*) and their average total job experience was 7.741 years (*S.E.* = *5.611 years*). Only 35.90% of these participants have a management position.

### 3.2. Measures

Since all the scales in our survey were initially developed in English, we translated them into Chinese according to the process of translation and back-translation (Brislin, 1980). First, the original scale was translated into Chinese by a bilingual professor. Then, another professor and two PhD. students (all bilingual) translated the Chinese scales back into English. Finally, they compared the translated scales to the originals and the four translators together discussed and resolved any minor translation issues. Unless specially noted, five-point Likert type scales ranging from 1 (strongly disagree) to 5 (strongly agree) were used in this study.

#### 3.2.1. EO REO

Four-item scale of EO REO is developed for this study. As Yoshikawa et al. (2020) published an REO scale during the middle of our research, we conducted an additional study to examine the convergent validity of our scale with Yoshikawa et al. (2020) scale. This supplementary work shows that EO REO measured by our scale and REO measured by Yoshikawa et al. (2020) scale have high convergent validity and the same nomological network. It offers support for the validity of our developed scale. For the details of the additional study, please see the Supplementary material. A sample item of our EO REO scale is “If the organization is willing to help employees when they need a special favor, employees should also help the organization achieve its goals.” Each participant rated their agreement on these opinions using five-point Likert type scales ranging from 1 (strongly disagree) to 5 (strongly agree). This scale yielded a Cronbach’s alpha reliability coefficient of 0.885.

#### 3.2.2. Voice behavior

Following recent research (e.g., Liu et al., 2013; Venkataramani et al., 2016), we used four items from Van Dyne and LePine’s (1998) six-item voice behavior scale. [Liu et al. (2013) argued that the two excluded items did not focus on verbal communication but only describe the general features of employees’ proactivity.] A sample item is “This employee develops and makes recommendations concerning issues that affect this organization.” The four-item scale yielded a Cronbach’s alpha reliability coefficient of 0.840.

#### 3.2.3. Challenge stressor

We used six items from Cavanaugh et al. (2000) to measure the situational presence of challenge stressors. Participants were asked to “indicate how much stress they have experienced on each of the six items” and a sample item was “the number of projects and or assignments I have.” The scale of challenge stressor in study 1 yielded a Cronbach’s alpha reliability coefficient of 0.909.

#### 3.2.4. Construal level

Eighteen items from Reyt and Wiesenfeld’s (2015) study were used to measure construal level. Each item described a common work activity and was followed by low construal level option and high construal level option. A sample work activity was “preparing a report.” The low-level description of this activity was “compiling information” and the high-level description of this activity was “showing progress.” For each of eighteen items, participants were asked to choose one option that best described how participants see the focal work activity. This scale yielded a Cronbach’s alpha reliability coefficient of 0.812. Following the recommendation of Vallacher and Wegner (1989), the number of high-level descriptions chosen was computed as the participants’ abstraction score.

#### 3.2.5. Control variables

Prior research suggests that gender, age, education, organizational tenure and job position might influence voice behavior (e.g., Van Dyne and LePine, 1998; Detert and Burris, 2007). For instance, Morrison (2011) has documented that there may be gender differences in voice and highly educated employees generally have more ideas to speak up (e.g., Liang et al., 2012). Moreover, experienced employees represented by age and organizational tenure may feel more comfortable to voice (e.g., Tangirala and Ramanujam, 2008), and employees with higher positions feel more responsible to speak up their concerns about the organization (e.g., Fuller et al., 2006). Therefore, gender was dummy-coded, with male participants coded as “0” and female participants coded as “1.” For education level, “high school and below” was coded as “1,” “junior college” was coded as “2” and “bachelor and above” was coded as “3.” Age and organizational tenure were self-reported in years. Job position was dummy-coded, with non-managers coded as “0” and managers coded as “1.” We used one-way analysis of variance (ANOVA) to examine the difference of voice behavior among different enterprises. The results indicated that there was no significant difference among these enterprises (*F[11, 225]* = *0.692, p* < *0.1*). Hence, enterprises were not controlled in the model.

### 3.3. Results and discussion

Table 1 shows the means, standard deviations, and correlation coefficients of the variables in study 1. We conducted a confirmatory factor analysis (CFA) to examine the construct validity of the above-described measures. We followed the recommendation of Vallacher and Wegner (1989) and reflected construal level as the sum of high-level descriptions chosen. As such, we first computed the scale score of construal level and loaded this scale score on the latent factor in the measurement model. The items of EO REO, voice behavior and challenge stressor were used as observed indicators in the confirmatory factor analysis. We examined the fit of the one-factor, two-factor (combining challenge stressor and construal level, combining EO REO and voice behavior), three-factor (challenge stressor, construal level and combining EO REO and voice behavior) against our hypothesized four-factor measurement model. Among all of these measurement models, the hypothesized four-factor measurement model (containing EO REO, voice behavior, challenge stressor and construal level) gave the best fit for the data (χ^2^ = *224.767, df* = *85, CFI* = *0.928, IFI* = *0.929, RMSEA* = *0.083*).

**TABLE 1:** The correlations between the variables in study 1.

Variables	1	2	3	4	5	6	7	8	9
1. EO REO	(0.885)								
2. Voice behavior	0.125	(0.840)							
3. Challenge stressor	-0.056	0.062	(0.909)						
4. Construal level	0.080	0.093	0.039	(0.812)					
5. ^a^Gender	0.066	-0.228***	-0.083	0.054	–				
6. Age in year	0.036	0.056	0.068	-0.019	-0.137*	–			
7. ^b^Education	-0.013	0.126	0.009	0.002	0.048	-0.244***	–		
8. Organizational tenure in year	-0.054	0.014	0.046	-0.015	-0.097	0.701***	-0.256***	–	
9. ^c^Position	-0.065	0.118	0.119	-0.019	-0.090	0.245***	-0.021	0.106	–
Mean	4.050	3.769	3.217	10.608	–	30.806	–	5.163	–
S.D.	0.951	0.904	0.929	4.080	–	5.569	–	4.696	–

*N* = 237, *p < 0.05, ***p < 0.001; EO REO represents employees-organization reciprocal exchange orientation. The values in the parentheses represent Cronbach’α reliability coefficient. ^a^Gender (“0” male; “1” female). ^b^Education (“1” high school and blow; “2” junior college; “3” bachelor and above). ^c^Position (“0” non-managers; “1” managers).

All the interaction items were computed using the centralized values of EO REO, challenge stressor and construal level. We used regression analysis to examine the three-way interaction model and results are shown in Table 2. As hypothesized, Model 2 shows that EO REO is positively related with voice behavior (*B* = *0.141, S.E.* = *0.060, p* < *0.05*) but the interaction between EO REO and challenge stressors is not (*B* = −*0.021, S.E.* = *0.055, p* > *0.05*). Hypothesis 1 is not supported. However, Model 5 shows a significant three-way interaction suggesting that the moderating effect of challenge stressor on the relationship between EO REO and voice behavior was affected by construal level (*B* = −*0.040, S.E.* = *0.019, p* < *0.05*).

**TABLE 2:** The results of hierarchical regression in study 1.

Variables	Model 1	Model 2	Model 3	Model 4	Model 5
Constant	3.105 (0.504)***	2.633 (0.538)***	2.489 (0.580)***	2.320 (0.593)***	2.393 (0.590)***
^a^Gender	−0.401 (0.115)***	−0.419 (0.115)***	−0.412 (0.115)***	−0.420 (0.115)***	−0.406 (0.115)***
Age in year	0.008 (0.015)	0.004 (0.015)	0.004 (0.015)	0.003 (0.015)	−0.002 (0.015)
^b^Education	0.267 (0.119)*	0.272 (0.118)*	0.276 (0.119)*	0.283 (0.120)*	0.297 (0.119)*
Organizational tenure in year	−0.003 (0.017)	0.002 (0.017)	0.002 (0.017)	0.004 (0.017)	0.007 (0.017)
^c^Position	0.171 (0.123)	0.195 (0.122)	0.183 (0.124)	0.182 (0.124)	0.200 (0.123)
EO REO		0.141 (0.060)*	0.145 (0.061)*	0.138 (0.061)*	0.138 (0.061)*
Challenge stressor			0.035 (0.062)	0.027 (0.063)	0.044 (0.063)
EO REO × challenge stressor			−0.021 (0.055)	−0.019 (0.056)	−0.008 (0.056)
Construal level				0.022 (0.014)	0.018 (0.014)
EO REO × construal level				−0.021 (0.017)	−0.018 (0.017)
Challenge stressor × construal level				0.001 (0.015)	0.010 (0.016)
EO REO × challenge stressor × construal level					−0.040 (0.019)*
F	4.150***	4.442**	3.368***	2.818**	2.991***
Adjust R^2^	0.063	0.080	0.074	0.078	0.092
ΔR^2^	–	0.021	0.002	0.015	0.017*

*N* = 237, *p < 0.05, **p < 0.01, ***p < 0. 001; EO REO represents employees-organization reciprocal exchange orientation. All data are unstandardized estimates and the values in the parentheses represent the standard error of the unstandardized regression coefficient. ^a^Gender (“0” male; “1” female). ^b^Education (“1” high school and blow; “2” college; “3” bachelor and above). ^c^Position (“0” non-managers; “1” managers).

**FIGURE 1:**
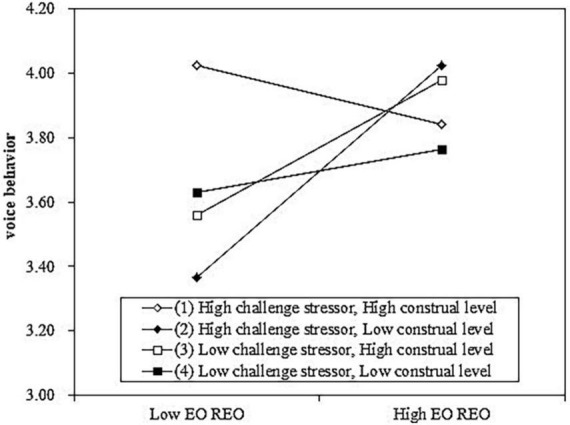
Results of the three-way interaction in study 1 [employees-organization reciprocal exchange orientation (EO REO) represents employees-organization reciprocal exchange orientation].

To further examine our hypothesis, we conducted simple slopes analysis and depicted the interaction using the excel sheets in Dawson’s website.^1^ We divided high and low values for both challenge stressor and construal level based on one standard deviation above and below the mean value of each variable (Aiken and West, 1991). Figure 1 depicted the interaction. For employees with low construal level, the positive relationship between EO REO and voice behavior is significant when challenge stressor is high (*Slope* = *0.347, S.E.* = *0.130, p* < *0.05*) and the positive relationship between EO REO and voice behavior is not significant when challenge stressor is low (*Slope* = *0.072, S.E.* = *0.124, p* > *0.1*). Moreover, for employees with high construal level, the relationship between EO REO and voice behavior is not significant when the challenge stressor is high (*Slope* = −*0.095, S.E.* = *0.128, p* > *0.1*) or low (*Slope* = *0.220, S.E.* = *0.129, p* > *0.05*). Therefore, hypothesis 2 was supported.

Study 1 therefore offers empirical support for the three-way interaction. However, the research design whereby one supervisor rated only one subordinate’s voice behavior has two major limitations. First, subordinates supervised by one immediate leader are very likely to engage in similar behavior, and they might all exhibit more or less voice behavior than subordinates supervised by another immediate leader. Second, it is possible that supervisors have their own subjective standard or bias in evaluating subordinates’ voice behavior. In order to address this concern, it is better to collect data using the research design whereby one supervisor rated several subordinates’ voice behavior and analyze data using a hierarchical linear model. We therefore retested our hypothesis and addressed the abovementioned limitation in study 2.

## 4. Study 2

### 4.1. Participants and procedures

We used the authors’ personal relationships to access 319 participants who are supervised by 61 direct leaders from the Chinese population. Similar to the collection procedure in study 1, we used two separate questionnaires: one for subordinates and the other for their immediate supervisors. Each subordinate completed a scale of EO REO, challenge stressors, construal level, and some personal information. Supervisors rated each subordinate’s voice behavior. The number of subordinates that one supervisor rated ranged from 3 to 7 (*Mean* = *5.230, S.E.* = *0.716*). Finally, we obtained valid and complete questionnaires from 225 subordinates who are supervised by 60 direct leaders.

Among these 225 participants who had matched immediate supervisors, over half (57.30%) of them were male and almost half (50.20%) of them were unmarried. The average age of these participants was 32.342 years (*S.E.* = *8.272 years*). In terms of education, 32.00% of the participants had bachelor’s degrees, 24.00% of them had master’s degrees and above, and the others had junior college’s degrees and lower levels of schooling. 46.20% of these participants had organizational tenure of less than 1 year (including 1 year), 34.70% of them had organizational tenure ranging from 1 to 2 years (including 2 years), 16.40% of them had organizational tenure ranging from 2 to 5 years (including 5 years), and the others had organizational tenure more than 5 years. For job experience, 14.20% of these participants had job experience of less than 1 year (including 1 year), 29.30% of them had job experience ranging from 1 to 2 years (including 2 years), 37.80% of them had job experience ranging from 2 to 5 years (including 5 years), and the others had job experience more than 5 years. Most of the participants had no management position (60.90%).

### 4.2. Measures

We translated the English scales using the same process in study 1. And unless specially noted, five-point Likert type scales ranging from 1 (strongly disagree) to 5 (strongly agree) were used in study 2.

#### 4.2.1. E-O REO

We used the same scale in study 1 to measure E-O REO and this scale yielded a Cronbach’s alpha reliability coefficient of 0.703 in study 2.

#### 4.2.2. Voice behavior

As in study 1, four items from Van Dyne and LePine’s (1998) study were used to measure voice behavior. This scale yielded a Cronbach’s alpha reliability coefficient of 0.717 in study 2.

#### 4.2.3. Challenge stressors

As in study 1, a six item scale of challenge stressors (Cavanaugh et al., 2000) was used to measure work stressors. It yielded a Cronbach’s alpha reliability coefficient of 0.706 in study 2.

#### 4.2.4. Construal level

As in study 1, eighteen items form Reyt and Wiesenfeld (2015) were used to measure construal level. This scale yielded a Cronbach’s alpha reliability coefficient of 0.879 in study 2.

#### 4.2.5. Control variables

Gender, age, education, organizational tenure, and job position were also chosen as control variables in study 2. The coding of gender and job position was the same as study 2. In addition, “junior college and blow” was coded as “1,” “bachelor” was coded as “2” and “master and above” was coded as “3.” For organizational tenure, “≤1 year” was coded as “1,” “>1 year and ≤2 years” was coded as “2,” “>2 years and ≤5 years” was coded as “3,” “>5 years” was coded as “4.”

### 4.3. Analytical approach

Different from Study 1, each supervisor provided ratings of voice for multiple employees. Therefore, the employees in our sample were nested within their supervisors and this violated the independence assumption. To address the non-independence in our dependent variable, we used multilevel data modeling (Raudenbush and Bryk, 2002) to test our hypothesis. Specifically, we used HLM (hierarchical linear model) version 6.08 with restricted maximum-likelihood (RML) estimation for the analysis. EO REO, challenge stressor, construal level and voice behavior were individual-level (level 1) variables and there were no group-level (level 2) predictors in the analysis. Following Hofmann et al.’s (2000) recommendation, we group centered all the level-1 variables when conducting the hierarchical regression.

### 4.4. Results and discussion

Table 3 shows the means, standard deviations and correlation coefficients of the variables in study 2. As in study 1, we conducted a confirmatory factor analysis first. Although the hypothesized four-factor measurement model (containing EO REO, voice behavior, challenge stressor and construal level) gave better fit for the data than any one-factor, two-factor and three-factor measurement models, the four-factor measurement model did not fit the data well (χ^2^ = *254.972, df* = *85, CFI* = *0.762, IFI* = *0.769, RMSEA* = *0.094*). Following the similar procedure of Tuckey et al. (2015) and Crane and Searle (2016), we therefore considered challenge stressors as a higher order factor indicated by three 2-item subscales. These challenge stressor subscales were workload (i.e., “The number of projects and or assignments I have” and “The amount of time I spend at work”), time pressure (i.e., “Time pressures I experience” and “The volume of work that must be accomplished in the allotted time”) and job responsibility (i.e., “The amount of responsibility I have” and “The scope of responsibility my position entails”). Similar to the procedure of Zhang et al. (2014), we computed the scale scores for each of these three subscales and loaded these three subscale scores into challenge stressors factor. By this approach, we reexamined the fit of the hypothesized four-factor measurement model and found a good fit to the data (χ^2^ = *92.567, df* = *49, CFI* = *0.907, IFI* = *0.910, RMSEA* = *0.063*).

**TABLE 3:** The correlations between the variables in study 2.

Variables	1	2	3	4	5	6	7	8	9
1. EO REO	(0.703)								
2. Voice behavior	-0.192**	(0.717)							
3. Challenge stressor	0.287***	-0.095	(0.706)						
4. Construal level	0.203**	-0.077	-0.032	(0.879)					
5. ^a^Gender	0.004	0.062	-0.086	0.027	–				
6. Age in year	-0.060	0.022	-0.071	0.068	-0.127	–			
7. ^b^Education	-0.088	-0.030	-0.077	-0.062	-0.004	0.117	–		
8. ^c^Organizational tenure in year	0.058	-0.040	-0.054	0.246***	-0.444***	0.399***	0.052	–	
9. ^d^Position	-0.098	-0.022	-0.042	0.015	-0.010	0.063	0.767***	0.172**	–
Mean	3.433	3.381	3.258	4.702	–	32.342	–	–	–
S.D.	0.853	0.855	0.655	4.492	–	8.272	–	–	–

*N* = 225, **p < 0.01, ***p < 0.001; EO REO represents employees-organization reciprocal exchange orientation. The values in the parentheses represent Cronbach’α reliability coefficient. ^a^Gender (“0” male; “1” female). ^b^Education (“1” junior college and blow; “2” bachelor; “3” master and above). ^c^Organizational tenure in year (“1” ≤1 year, “2”>1 year and ≤2 years, “3”>2 years and ≤5 years, “4”>5 years), ^d^Position (“0” non-managers; “1” managers).

We used a one-way analysis of variance (ANOVA) with voice as the dependent variable to examine the non-independence of supervisor-rated voice behavior. The results indicated that, as expected, each supervisor rated their employees on voice in significantly different ways (*F[59, 165]* = *4.740, p* < *0.001; ICC[1]* = *0.495*). This provided evidence that modeling supervisor-rated voice as non-independent was both appropriate and necessary. Hence, we used HLM to further examine our hypotheses.

We conducted hierarchical moderated regression analysis to examine the three-way interaction model and results are shown in Table 4. Model 3 shows that the positive relationship between EO REO and voice behavior is not significant (*B* = *0.022, S.E.* = *0.062, p* > *0.1*), nor is the interaction between EO REO and challenge stressors (*B* = −*0.075, S.E.* = *0.094, p* > *0.05*). Hypothesis 1 is not supported in study 2. Hypothesis 2 is tested in Model 6 which shows that the moderating effect of challenge stressor on the relationship between EO REO and voice behavior was moderated by construal level (*B* = −*0.037, S.E.* = *0.019, p* < *0.05*).

**TABLE 4:** The results of hierarchical linear model in study 2.

Variables	Model 1	Model 2	Model 3	Model 4	Model 5	Model 6
Intercept	3.355 (0.087)***	3.355 (0.087)***	3.355 (0.087)***	3.355 (0.087)***	3.355 (0.087)***	3.355 (0.087)***
^a^Gender		0.174 (0.096)^+^	0.173 (0.098)^+^	0.169 (0.095)^+^	0.151 (0.097)	0.146 (0.095)
Age in year		0.005 (0.007)	0.005 (0.007)	0.005 (0.007)	0.005 (0.006)	0.005 (0.006)
^b^Education		−0.091 (0.093)	−0.094 (0.090)	−0.118 (0.088)	−0.084 (0.088)	−0.086 (0.086)
^c^Organizational tenure in year		0.023 (0.071)	0.022 (0.072)	0.016 (0.071)	0.009 (0.067)	0.016 (0.064)
^d^Position		0.069 (0.145)	0.076 (0.139)	0.124 (0.157)	0.058 (0.162)	0.063 (0.153)
EO REO			0.022 (0.062)	0.014 (0.069)	0.016 (0.069)	0.008 (0.068)
Challenge stressor				−0.058 (0.073)	−0.042 (0.071)	−0.005 (0.077)
EO REO × challenge stressor				−0.075 (0.094)	−0.132 (0.102)	−0.098 (0.112)
Construal level					0.014 (0.018)	0.027 (0.019)
EO REO × construal level					−0.026 (0.012)*	−0.033 (0.012)**
Challenge stressor × construal level					0.033 (0.017)^+^	0.059 (0.019)**
EO REO × challenge stressor × construal level						−0.037 (0.019)*
σ^2^	0.3679	0.3698	0.3719	0.3716	0.3654	0.3589
τ (intercept)	0.3604	0.3599	0.3593	0.3594	0.3610	0.3627
−2 log likelihood	507.167	521.145	526.409	530.511	542.177	546.289

*N* (level 1) = 225, *N* (level 2) = 60; ^+^*p* < 0.1, *p < 0.05, **p < 0.01, ***p < 0. 001; EO REO represents employees-organization reciprocal exchange orientation. All data are unstandardized estimates and the values in the parentheses represent the standard error of the unstandardized regression coefficient. ^a^Gender (“0” male; “1” female). ^b^Education (“1” junior college and blow; “2” bachelor; “3” master and above). ^c^Organizational tenure in year (“1” ≤1 year, “2”>1 year and ≤2 years, “3”>2 years and ≤5 years, “4”>5 years), ^d^Position (“0” non-managers; “1” managers).

**FIGURE 2:**
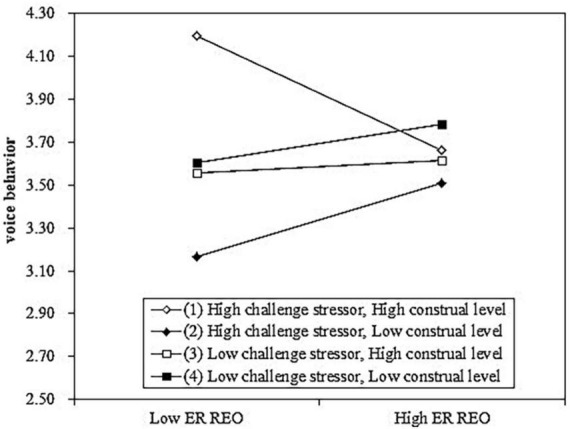
Results of the three-way interaction in study 2 [employees-organization reciprocal exchange orientation (EO REO) represents employees-organization reciprocal exchange orientation].

To further examine our hypothesis, we also conducted simple slopes analysis and depicted the interaction (see Figure 2). For employees with low construal level, the positive relationship between EO REO and voice behavior is marginally significant when challenge stressor is high (*Slope* = *0.205, S.E.* = *0.126, p* < *0.1*) and the positive relationship between EO REO and voice behavior is not significant when challenge stressor is low (*Slope* = *0.109, S.E.* = *0.128, p* > *0.1*). For employees with high construal level, the relationship between EO REO and voice behavior is significantly negative when challenge stressor is high (*Slope* = −*0.310, S.E.* = *0.128, p* < *0.05*) and the positive relationship between EO REO and voice behavior is not significant when challenge stressor is low (*Slope* = *0.040, S.E.* = *0.127, p* < *0.1*). Therefore, hypothesis 2 was again supported in study 2.

## 5. Conclusion and discussion

In this research, we explored when employees with EO REO will speak up and use construal level theory to underpin the three-way interaction. Our results were generally supported for the conditional positive impact of EO REO on voice behavior. More specifically, the results showed that, for employees with low construal level, challenge stressors can strengthen the positive relationship between EO REO and voice behavior but for employees with high construal level, challenge stressors are problematic for those with high EO REO. These findings offer important theoretical implications for understanding when employees with high levels of EO REO utilize voice to exchange resource with their organizations.

### 5.1. Theoretical implications

This study extends the personality approach of voice behavior by addressing the role of EO REO. Because of the positive nature of voice behavior in organizational function (Van Dyne and LePine, 1998), it is important for scholars and managers to understand which kind of personal characteristic is associated with more frequent voice behavior. Previous research has offered answers about the predicting role of Big Five factors of personality (i.e., openness to experience, agreeableness, conscientiousness, extraversion, and neuroticism) and proactivity personality (e.g., Avery, 2003; Chamberlin et al., 2017; Xu et al., 2019; Li and Xu, 2020), but known little about whether employee’s orientation in favor for the norm of reciprocity can be an antecedent of voice behavior.

This study goes beyond main effects of personality by offering a boundary condition about when employees’ personal belief in reciprocity norm has more accurate prediction on their voice behavior. Indeed, the main effect relationship between EO REO and voice behavior appears to not be very stable, as we found a significant relationship in study 1 but not in study 2. This finding is consistent with Yoshikawa et al. (2020) study that REO is not significantly related with organizational citizenship behavior. Yoshikawa et al. (2020) argue that the reason responsible for this is that the impact of REO on reciprocity-related behavior is in accordance with the quality of the interpersonal relationship. Findings of our study extend our knowledge about the null effect of REO. Both studies show that whether employees engage in voice behavior as a reaction to their high EO REO is only emerged when challenge stressor is high but construal level is low. This indicates that the positive association between EO REO and voice behavior is jointly moderated by challenge stressor and construal level. Further, findings of the three-way interaction also illustrate that a deeper understanding about the relationship between individual difference (e.g., EO REO) and behavior (e.g., voice behavior) should consider not only the situation (e.g., challenge stressor) but also employees’ mindset regarding that situation (e.g., construal level).

This study deepens our knowledge that employees who comply with the reciprocal norm should reciprocate with pro-organizational behavior when they are faced with challenge stressors. Challenge stressors are a kind of eustress which has the probability to promote personal growth (Cavanaugh et al., 2000). As such, it is straightforward to assume that employees with high-level EO REO are most likely to engage in voice behavior in the face of challenge stressors. However, we found that the positive impact of EO REO on voice behavior was not established when challenge stressor is high and construal level is high. More specifically, study 1 found a non-significant relationship and study 2 found a negative relationship. It is because challenge stressors require employees with a high construal level mindset to focus on the details of the job, which is contrary to their preferred abstract mindset. Accordingly, those employees may be dissatisfied with high challenge stressors and respond negatively. Moreover, we found that EO REO has a positive effect on voice behavior when challenge stressor is high and construal level is low. This finding supports our assumption that the style of coping with challenge stressor is more aligned with the mindset of employees who have low construal level. Thus, those employees with high EO REO will reciprocate positively. These findings indicate that construal level may determine how people cope with challenge stressor, which is consistent with Han et al.’s (2016) theory that the two strategies of problem-focused and emotion-focused coping are associated with lower and higher construal levels, respectively.

### 5.2. Limitations and future study

Although we examined our hypothesis using a multi-source research design, there are some limitations that should be considered in future studies. First, we did not obtain a robust result for the positive relationship between EO REO and voice behavior. It indicates that there are some moderators which can regulate such relationship. Although we have found the jointly moderating effect of challenge stressor and construal level, the effect size is small. Hence, further study should explore other moderators and examine the impact of EO REO on voice behavior in other situations. For example, perceived organizational support refers to employees’ subjective perceptions about whether organizations pay attention to their contributions and well-being (Rhoades and Eisenberger, 2002). When perceiving organizational support, those employees with high EO REO might be more likely to engage in voice behavior as a way of reciprocating. Voice climate is defined as employees’ shared perceptions about voice behavior (Frazier and Bowler, 2015). Employees in the situation with a strong voice climate have a high tendency to believe that voice behavior is allowed in the organization (Frazier and Bowler, 2015). Therefore, employees with high EO REO are more likely to utilize voice behavior and reciprocate with their organizations.

Second, the cross-sectional data used in our study are not conducive to inferring causal relationships. However, EO REO is a kind of individual characteristics which is more stable (Yoshikawa et al., 2020). Thus, we believe that the influence of EO REO on voice behavior is easier than the reverse effect. Moreover, our study examined a three-way interaction and it is not sensitive to the cross-sectional data (Aguinis et al., 2005). In spite of this, future studies can still adopt longitudinal, experimental designs to comprehensively rule out potential reverse causality.

Third, as previous studies have noted, sample characteristics may influence the result of the interaction effect (Aguinis et al., 2005). In particular, the generalizability of the results may be constrained due to the fact that our samples come from enterprises in China. As high power distance is a feature of Chinese culture (Farh et al., 1997), voice behavior in terms of challenging the authority is not encouraged in such hierarchical culture. As a consequence, our findings may be more applicable in the organizations with hierarchical culture. Hence, future research should examine the findings of our study in different contexts.

### 5.3. Practical implications and conclusion

Our study shows that employees whose EO REO is high are more willing to speak out their ideas about organizational development. According to this finding, EO REO might be a valuable characteristic that managers should consider when recruiting employees. Furthermore, we found that the relationship between EO REO and voice behavior is more likely to be positive when employees with low construal level are suffering from challenge stressor. In modern organizations, challenging job demands are very pervasive (Lu, 1997; Casey, 2012; American Psychological Association, 2018). To increase voice behavior, it is important for managers to encourage employees to develop a high EO REO but also to decrease employees’ construal level when dealing with stressors. Our findings that the positive relationship between EO REO and voice behavior is not established when both challenge stressor and construal level are high indicates employees whose construal level is high may not adapt to working under high challenge stressor. Thus, it is a better practice to help those employees reduce challenge stressor or improve their ability of using the problem-focused coping style.

In conclusion, our study indicated that EO REO had a beneficial effect on voice behavior depending upon the variations of challenge stressors and employees’ construal level. The positive relationship between EO REO and voice behavior is only established when challenge stressor is high but construal level is low. The findings of our study provide more answers about who and when employees will speak up to benefit their organization.

## Data availability statement

The raw data supporting the conclusions of this article will be made available by the authors, without undue reservation.

## Ethics statement

The studies involving human participants were reviewed and approved by Hohai University. The patients/participants provided their written informed consent to participate in this study.

## Author contributions

LC, KU, and LZ contributed to conception and design of the study. LC wrote the first draft of the manuscript, performed the statistical analysis, and recipient of the fund. KU critically revised the manuscript. LZ organized the database. All authors contributed to manuscript revision, read, and approved the submitted version.

## Footnotes

1
http://www.jeremydawson.com/slopes.htm
