# Supplementary material for: Reciprocal exchange orientation to organization, challenge stressor and construal level: Three-way interaction effects on voice behavior
Source: Front Psychol. 2023 Feb 14;14:1119596. doi: 10.3389/fpsyg.2023.1119596 (PMC9971230; doi:10.3389/fpsyg.2023.1119596)
Supplement: Supplementary file 1 [file Data_Sheet_1.docx]

Supplementary Material

Reciprocal Exchange Orientation to Organization, Challenge Stressor and Construal Level: Three-Way Interaction Effects on Voice Behavior

Long Chen^1^, Kerrie Unsworth^2^, Li Zhang^3^

*** Correspondence:** Long Chen: chenlong4845@163.com

# Questionnaire (Study 1 and Stud 2)

## Employees-Organization Reciprocal Exchange Orientation

1. If the organization is willing to help employees when they need a special favor, employees should also help the organization achieve its goals.

2. If the organization considers employees’ goals and values, employees should regard achieving organizational goals as personal obligation.

3. If the organization values employees’ contribution, employees should also do extra work that is helpful for organization.

4. If the organization cares employees’ well-beings, employees should also care about organizational further development.

## Voice Behavior

1. This employee develops and makes recommendations concerning issues that affect this organization.

2. This employee speaks up and encourages others in this group to get involved in issues that affect this organization.

3. This employee communicates his/her opinions about work issues to others this organization even if his/her opinion is different and others this organization disagree with him/her.

4. This employee speaks up in this organization with ideas for new projects or changes in procedures.

## Challenge Stressor

1. The number of projects and or assignments I have.

2. The amount of time I spend at work.

3. The volume of work that must be accomplished in the allotted time.

4. Time pressures I experience.

5. The amount of responsibility I have.

6. The scope of responsibility my position entails.

## Construal Level

| **Item** | **Low-Level Description** | **High Level Description** |
| --- | --- | --- |
| **Preparing a report** | Compiling information | Showing Progress |
| **Using a computer** | Typing on a keyboard | Processing information |
| **Filling out a business form** | Filling in blanks with information | Following work protocol |
| **Obtaining information from someone** | Asking relevant questions | Gaining knowledge |
| **Making a presentation** | Presenting relevant material | Communicating knowledge |
| **Assigning work to someone** | Telling someone what to do | Getting things done |
| **Communicating information to someone** | Sending an email or talking to someone | Keeping someone informed |
| **Analyzing a data set** | Comparing numbers | Identifying trends |
| **Attending a meeting** | Being present and paying attention | Staying up to date |
| **Developing a procedure** | Writing down step-by-step instructions | Increasing work efficiency |
| **Writing business correspondence** | Composing an email | Maintaining a good business relationship |
| **Hiring someone** | Interviewing candidates | Maintaining staff level |
| **Developing a budget** | Listing expenses and revenues | Managing funds |
| **Proofreading a document** | Reading carefully for errors | Ensuring accuracy |
| **Training someone** | Showing someone how to do things | Increasing someone’s productivity |
| **Analyzing an operational report** | Reviewing information | Ensuring smooth operation |
| **Orienting a new worker** | Showing a new worker around | Acclimating a new worker |
| **Evaluating someone’s performance** | Reviewing quality of work | Providing feedback |

# Pilot Study

In our study, employees-organization reciprocal exchange orientation (i.e., EO REO) refers to employees’ personal belief that their exchange with organizations depends on the norm of reciprocity. Following the structure of Yoshikawa’s (2017) reciprocal exchange orientation scale, we adapted items of Hayton et al.’s (2012) scale to refine perceived organizational support and the form of repaying organization comes from the Eisenberger et al.’s (2001) felt obligation scale. The items used are “If the organization is willing to help employees when they need a special favor, employees should also help the organization achieve its goals”, “If the organization considers employees’ goals and values, employees should regard achieving organizational goals as personal obligation”, “If the organization values employees’ contribution, employees should also do extra work that is helpful for organization” and “If the organization cares employees’ well-beings, employees should also care about organizational further development”.

To validate our measure of EO REO, we compare it with Yoshikawa’s et al. (2020) REO scale and examine the convergent validity between these two scales. Further, we also include scale of negotiated exchange orientation (NEO) and generalized exchange orientation (GEO). By doing this, we can demonstrate that both our scale and Yoshikawa’s et al. (2020) REO scale have same nomological network.

## Participants and Procedures

We assessed 300 participants via a website named Questionnaire Star (https://www.wjx.cn/) and obtained 266 completed responses. All participants were asked to complete EO REO, NEO, GEO and other demographic variables. Among these 266 participants, over half (53.40%) of them were female and more than half of them (66.50%) were married. For age, half of them (52.30%) were between 25 and 34, 26.70% of them were between 35 and 59, 19.90% of them were between 18 and 24, 0.40% of them were above 60 and the others were below 17. In terms of education, 75.90% of the participants had bachelor’s degrees, 10.50% of them had master’s degrees and above, and the others had junior college’s degrees and lower levels of schooling. 12.00% of these participants had organizational tenure of less than 1 year (including 1 year), 30.50% of them had organizational tenure ranging from 1 to 2 years (including 2 years), 28.20% of them had organizational tenure ranging from 2 to 5 years (including 5 years), and the others had organizational tenure more than 5 years. For job experience, 11.30% of these participants had job experience of less than 1 year (including 1 year), 21.40% of them had job experience ranging from 1 to 2 years (including 2 years), 20.30% of them had job experience ranging from 2 to 5 years (including 5 years), and the others had job experience more than 5 years. Most of the participants had management position (57.50%).

## Measures

Since all the scales in our survey were initially developed in English, we translated them into Chinese according to the process of translation and back-translation (Brislin, 1980). First, the original scale was translated into Chinese by a bilingual professor. Then, another professor and two PhD students (all bilingual) translated the Chinese scales back into English. Finally, they compared the translated scales to the originals and the four translators together discussed and resolved any minor translation issues. Unless specially noted, five-point Likert type scales ranging from 1 (strongly disagree) to 5 (strongly agree) were used in this study.

***EO REO.*** Four items developed in our study were used to measure EO ERO. Each participant rated their agreement on the opinions using five-point Likert type scales ranging from 1 (strongly disagree) to 5 (strongly agree). This scale yielded a Cronbach's alpha reliability coefficient of 0.728. We also measured employees’ EO REO using Yoshikawa’s et al. (2020) four-item REO scale and changed the targets into “organization”. A sample item is “When I receive support from the organization, I should remember to give something back to the organization”. Each participant rated their agreement on these opinions using five-point Likert type scales ranging from 1 (strongly disagree) to 5 (strongly agree). This scale yielded a Cronbach's alpha reliability coefficient of 0.741.

***NEO.*** Four items from Yoshikawa’s et al. (2020) study were used to measure NEO and we also changed the targets into “organization”. A sample item is “At work, it generally pays to clarify rewards before making extra efforts for the organization”. Each participant rated their agreement on these opinions using five-point Likert type scales ranging from 1 (strongly disagree) to 5 (strongly agree). This scale yielded a Cronbach's alpha reliability coefficient of 0.777.

***GEO.*** Twelve items from Yoshikawa’s et al. (2020) study were used to measure GEO. Four items were used to measure unilateral giving with an expectation of indirect reciprocation (UG). A sample item is “My efforts for colleagues will be rewarded by someone at some point, if not immediately”. Four items were used to measure paying it forward (PIF). A sample item is “When someone in the workplace makes extra efforts for me, I often start thinking what I can do for others”. Four items were used to measure rewarding reputation (RR). A sample item is “When a colleague who often gives support to others is in trouble, I should do something for him/her”. Each participant rated their agreement on these opinions using five-point Likert type scales ranging from 1 (strongly disagree) to 5 (strongly agree). The Cronbach's alpha reliability coefficient of UG, PIF and RR is 0.678, 0.704 and 0.727 respectively.

## Results

To examine whether items of our EO REO and items of Yoshikawa’s et al. (2020) scale are measuring the same concept, we conducted confirmatory factor analysis (CFA). First, we loaded eight items of EO REO into one factor and the one-factor measurement model gave the best fit for the data (*χ^2^ = 24.835, df = 20, CFI = 0.992, IFI= 0.992, RMSEA = 0.030*). Factor loadings are large (range: 0.554 to 0.656) and significant (*t > 2.00*), the composite reliability is acceptable (0.839), supporting convergent validity (Fornell & Larcker, 1981). These results indicate that items from our scale and Yoshikawa’s et al. (2020) scale can reflect one factor. Second, we also conducted a two-factor CFA by loading four items of our scale as one factor and four items of Yoshikawa’s et al. (2020) scale on one factor. Results indicates that the two-factor measurement model gave the best fit for the data (*χ^2^ = 19.384, df = 19, CFI = 0.999, IFI= 0.999, RMSEA = 0.001*). For our scale, factor loadings are large (range: 0.569 to 0.683) and significant (*t > 2.00*), the composite reliability is acceptable (0.731), supporting convergent validity (Fornell & Larcker, 1981). For Yoshikawa’s et al. (2020) scale, factor loadings are large (range: 0.619 to 0.658) and significant (*t >2.00*), the composite reliability is acceptable (0.743), supporting convergent validity (Fornell & Larcker, 1981). More importantly, the average variance extracted (AVE) for each construct is smaller than the square of correlations between constructs. These results indicate that both of these two scales have their own convergent validity, but they are not discriminative, supporting that our scale and Yoshikawa’s et al. (2020) scale are measuring one concept.

Before exploring the nomological network, we conducted CFA and the hypothesized six-factor measurement model (containing our scale of EO REO, Yoshikawa’s et al. (2020) scale of REO, NEO, UG, PIF and RR) gave the best fit for the data (*χ^2^ = 467.697, df = 237, CFI = 0.905, IFI= 0.906, RMSEA = 0.061*). The means, standard deviations and correlation coefficients of these variables are showed in Table 1. Table 1 demonstrates that EO REO measured by our scale and REO measured by Yoshikawa’s et al. (2020) scale are correlated with NEO (*EO REO: r=0.153, p<0.05; REO: r=0.173, p<0.05), UG (EO REO: r=0.665, p<0.001; REO: r=0.726, p<0.001*), PIF (*EO REO: r=0.688, p<0.001; REO: r=0.760, p<0.001*) and RR (*EO REO: r=0.658, p<0.001; REO: r=0.739, p<0.001*) in the same direction and similar strength.

Table 1 The correlations between the variables

| Variables | 1 | 2 | 3 | 4 | 5 | 6 |
| --- | --- | --- | --- | --- | --- | --- |
| 1. ^a^EO REO | (0.728) |  |  |  |  |  |
| 2. ^b^REO | 0.671*** | (0.741) |  |  |  |  |
| 3. NEO | 0.153* | 0.173* | (0.777) |  |  |  |
| 4. UG | 0.665*** | 0.726*** | 0.200** | (0.678) |  |  |
| 5. PIF | 0.688*** | 0.760*** | 0.158** | 0.720*** | (0.704) |  |
| 6. RR | 0.658*** | 0.739*** | 0.157* | 0.708*** | 0.761*** | (0.727) |
| Mean | 4.040 | 4.078 | 3.439 | 4.010 | 4.038 | 4.061 |
| S.D. | 0.614 | 0.624 | 0.875 | 0.589 | 0.627 | 0.637 |

Note: N=266, *p < 0.05, **p < 0.01, ***p < 0.001; The values in the parentheses represent cronbach’α reliability coefficient. ^a^EO REO is developed in our study. ^b^REO is from Yoshikawa’s et al. (2020) study.

Above results confirm that the new developed scale and Yoshikawa’s et al. (2020) scale have high convergent validity and same nomological network. Thus, the EO REO scale in present study can measure the same concept with Yoshikawa’s et al. (2020) scale.

## Reference

Brislin, R. W. (1980). Translation and content analysis of oral and written material. In H. C. Triandis (Ed.), Handbook of cross-cultural psychology (pp. 349-444). Boston: Allyn and Bacon.

Eisenberger, R., Armeli, S., Rexwinkel, B., Lynch, P. D., & Rhoades, L. (2001). Reciprocation of perceived organizational support. Journal of Applied Psychology, 86(1), 42-51. doi: 10.1037/0021-9010.86.1.42.

Fornell, C., & Larcker, D. F. (1981). Structural equation models with unobservable variables and measurement error: algebra and statistics. Journal of Marketing Research, 18(1), 39-50. doi: 10.2307/3151312.

Hayton, J. C., Carnabuci, G., & Eisenberger, R. (2012). With a little help from my colleagues: a social embeddedness approach to perceived organizational support. Journal of Organizational Behavior, 33(2), 235–249. doi: 10.1002/job.755.

Yoshikawa, K. (2017). Generalised exchange orientation: a new construct and its antecedents and consequences (Doctoral dissertation, The London School of Economics and Political Science (LSE)).

Yoshikawa, K., Wu, C. H., & Lee, H. J. (2020). Generalized exchange orientation: conceptualization and scale development. Journal of Applied Psychology, 105(3), 294-311. doi: 10.1037/apl0000438.
